# Haploinsufficiency of Akt1 Prolongs the Lifespan of Mice

**DOI:** 10.1371/journal.pone.0069178

**Published:** 2013-07-30

**Authors:** Aika Nojima, Masakatsu Yamashita, Yohko Yoshida, Ippei Shimizu, Harumi Ichimiya, Naomi Kamimura, Yoshio Kobayashi, Shigeo Ohta, Naoaki Ishii, Tohru Minamino

**Affiliations:** 1 Department of Cardiovascular Medicine, Chiba University Graduate School of Medicine, Chiba, Japan; 2 Kazusa DNA Research Institute, Kisarazu, Chiba, Japan; 3 Department of Biochemistry and Cell Biology, Institute of Development and Aging Sciences, Graduate School of Medicine, Nippon Medical School, Nakahara-ku, Kawasaki, Kanagawa, Japan; 4 Department of Molecular Life Science, Basic Medical Science and Molecular Medicine, Tokai University School of Medicine, Isehara, Japan; 5 Department of Cardiovascular Biology and Medicine, Niigata University Graduate School of Medical and Dental Sciences, Niigata, Japan; 6 PRESTO, Japan Science and Technology Agency, Kawaguchi, Saitama, Japan; Rutgers New Jersey Medical School, United States of America

## Abstract

There is increasing evidence that nutrient-sensing machinery is critically involved in the regulation of aging. The insulin/insulin-like growth factor-1 signaling pathway is the best-characterized pathway with an influence on longevity in a variety of organisms, ranging from yeast to rodents. Reduced expression of the receptor for this pathway has been reported to prolong the lifespan; however, the underlying mechanisms are largely unknown. Here we show that haploinsufficiency of *Akt1* leads to an increase of the lifespan in mice. *Akt1* ^+/–^ mice had a lower body weight than their littermates with less fat mass and normal glucose metabolism. Ribosomal biogenesis and the mitochondrial DNA content were significantly reduced in these mice, along with a decrease of oxidative stress. Consistent with the results obtained in mice, inhibition of Akt-1 promoted longevity in nematodes (*Caenorhabditis elegans*), whereas activation of Akt-1 shortened the lifespan. Inhibition of Akt-1 led to a decrease of ribosomal gene expression and the mitochondrial DNA content in both human cells and nematodes. Moreover, deletion of ribosomal gene expression resulted in a decrease of the mitochondrial DNA content and normalized the lifespan shortened by Akt-1 activation in nematodes. These results suggest that an increase of mitochondrial amount and energy expenditure associated with enhanced protein synthesis accelerates both aging and the onset of age-associated diseases.

## Introduction

Endocrine signaling was first linked to longevity when it was shown that mutations of *daf-2*, a homologue of the mammalian insulin/insulin-like growth factor-1 (IGF-1) receptor [1], dramatically prolonged the lifespan of the nematode *Caenorhabditis elegans* [2]. Genetic analysis subsequently demonstrated that reduction-of-function mutations affecting various genes in the insulin/IGF-1/phosphatidylinositol-3 kinase (PI3K)/Akt signaling pathway prolonged the lifespan of *C. elegans* [2]–[9]. Inhibiting this pathway confers longevity through changes in the expression of genes regulated by transcription factors such as the forkhead transcription factor DAF-16, the heat-shock transcription factor HSF-1, and the xenobiotic factor SKN-1 [10]. It has also been reported that the genes regulating longevity are conserved in a wide range of organisms ranging from yeast to mice. Mutation of *Sch9*, which is homologous with *Akt*, extends the lifespan of yeast [11], while mutations that decrease the activity of insulin/IGF-1 pathway improve the longevity of fruit flies [12] and mice [13], [14].

Target of rapamycin (TOR) is an evolutionarily conserved nutrient-sensing protein kinase that regulates growth and metabolism in all eukaryotic cells [15]. Studies performed in worms, flies, yeast, and mice support the notion that the TOR signaling network modulates aging [15]–[19]. Like inhibition of the insulin/IGF-1 pathway, inhibition of TOR increases resistance to environmental stress and requires transcriptional changes in order to extend the lifespan of yeast and worms [10], [20], [21]. In response to the intake of nutrients, TOR up-regulates translation activity, partly by activating the ribosomal subunit S6 kinase and inhibiting 4E-BP, which is a translation inhibitor. When nutrient levels and TOR activity are decreased, translation activity also declines. This appears to have a positive impact on the lifespan, since inhibition of S6 kinase improves longevity in yeast, nematodes, flies, and mice [10], [15], [22]. In *C. elegans*, DAF-16 plays an essential role in longevity related to inhibition of the insulin/IGF-1 pathway [23], [24], while inhibition of TOR extends the lifespan independently of DAF-16 [17], [25]. In mammals, however, the role of TOR in longevity related to inhibition of the insulin/IGF-1 pathway is largely unknown.

Here we studied *Akt1* ^+/–^ mice and found that their lifespan was significantly longer than that of littermates controls. We then sought to elucidate the mechanisms related to the increased longevity of these mice. *Akt1* ^+/–^ mice showed a decrease of TOR signaling, but phosphorylation of the forkhead transcription factors (FOXO) was not down-regulated. Gene ontology analysis suggested a crucial role of the suppression of translation and mitochondrial activity in promoting the longevity of *Akt1* ^+/–^ mice, suggesting that the TOR pathway is critically involved in prolonging the lifespan of mammals by inhibiting the insulin/IGF-1 pathway.

## Materials and Methods

### Animal Models

All experiments using live mice were performed in strict accordance with the guidelines of the American Association for Accreditation of Laboratory Animal Care, and the study protocol was approved by Chiba University Institutional Animal Care and Use Committee. *Akt1*-deficient mice (*Akt1* ^+/–^) were a kind gift from Dr. Morris J. Birnbaum (University of Pennsylvania School of Medicine, Philadelphia, PA). Generation and genotyping of *Akt1*-deficient mice have been described previously [26]. Heterozygous mice were backcrossed with wild-type C57BL/6 mice (SLC, Japan) for 6 generations. All mice were maintained under specific-pathogen-free conditions, and their lifespan was monitored by experienced technicians at Sankyo Laboratory Service (n = 101 for wild-type male mice, n = 103 for *Akt1* ^+/–^ male mice, n = 79 for wild-type female mice, n = 80 for *Akt1* ^+/–^ female mice). Survival curves were plotted by the Kaplan–Meier method, and differences between groups were evaluated by the log-rank test. The maximum lifespan was calculated as the average for the oldest 20% of the mice within each group [27]. For evaluation of the incidence of malignancy, 2-year-old mice were subjected to histopathologic examination by an experienced pathologist (Narabyouri Research Co., Ltd., Japan).

### Cell Culture and Retroviral Infection

Because it was very difficult to expand primary cultures of hepatocytes for infection, we utilized human endothelial cells that are highly proliferative. Human umbilical vein endothelial cells were purchased from Lonza (Walkersville, MD), and cultured according to the manufacturer’s instructions. We created a pLNCX (Clontech, Palo Alto, CA)-based vector expressing a dominant-negative form of AKT1 (AKTDN). Retroviral stocks were generated by transient transfection of a packaging cell line (293T, Clontech) and were stored at −80°C until use. Human endothelial cells (passages 4–6) were plated at 5×10^5^ cells in 100 mm diameter dishes at 24 hours before infection. Then the culture medium was replaced by retroviral stock supplemented with 8 mg/ml polybrene (Sigma, Tokyo, Japan) for infection. After 48 hours, infected cell populations were selected by culture in 500 mg/ml G418 for 7 days. On the 8th day post-infection, 1–3×10^5^ infected cells were seeded onto 100 mm diameter dishes. Oxygen consumption rates of cell cultures were determined with a 96-well BD Oxygen Biosensor System plate (BD Biosciences, San Jose, CA).

### Physiological Analysis

We housed mice individually to monitor their body weight and food intake. Adiposity was examined by CT scanning (LaTheta, Aloka) according to the manufacturer’s protocol. We obtained CT scans at 2 mm intervals from the diaphragm to the floor of the pelvic cavity. Oxygen consumption was measured in 8-week-old and 40-week-old mice with an O_2_/CO_2_ metabolic measurement system (Model MK-5000, Muromachikikai), as described previously [28]. Measurement of core body temperature and activity were performed as described previously [29].

### Laboratory Tests

For the intraperitoneal glucose tolerance test (IGTT), mice were starved for 6 hours and were given glucose intraperitoneally at a dose of 2 g kg^–1^ (body weight) in the early afternoon. For the insulin tolerance test, mice were given human insulin intraperitoneally (1 U kg^–1^ body weight) at 1: 00 pm without starvation. Tail vein blood was collected at 0, 15, 30, 60, and 120 minutes after administration and blood glucose levels were measured with a glucose analyzer (Roche Diagnostics). Plasma insulin levels were measured by immunoassay (Morinaga, Japan). Urinary 8-isoprostane was measured by a competitive assay (Japan Institute for the Control of Aging, Tokyo, Japan). Plasma levels of cholesterol, triglycerides, and free fatty acids were measured with commercial kits (Wako, Japan).

### Western Blot Analysis

Whole cell lysates were prepared in lysis buffer (10 mM Tris/HCl, pH 8, 140 mM NaCl, 5 mM EDTA, 0.025% NaN_3_, 1% Triton X-100, 1% deoxycholate, 0.1% SDS, 1 mM PMSF, 5 μg/ml leupeptin, 2 μg/ml aprotinin, 50 mM NaF, and 1 mM Na_2_VO_3_). Then the lysates (40–50 μg) were resolved by SDS-PAGE and proteins were transferred to a PVDF membrane (Millipore). The membrane was incubated with the primary antibody, followed by incubation with anti-rabbit or anti-mouse immunoglobulin-G conjugated with horseradish peroxidase (Jackson), and target proteins were detected by enhanced chemiluminescence (Amersham). The primary antibodies used for Western blotting were as follows: anti-phospho-mTOR (Ser2448) antibody (Cell Signaling Technology, #2971S), anti-phospho-p70 S6 kinase (Ser371) antibody (Cell Signaling Technology, #9208), anti-mTOR antibody (Cell Signaling Technology, #2983), anti-p70 S6 kinase antibody (Santa Cruz, sc-230), anti-phospho-FoxO3a antibody (Upstate), anti-FoxO3a antibody (Upstate).

### RNA Analysis

Total RNA was isolated from the livers of mice and from cultured cells with an RNeasy lipid tissue mini kit (Qiagen). For isolation of total RNA from C. elegans, an RNeasy MinElute Cleanup kit (Qiagen) was used. Real-time PCR was performed with a Light Cycler (Roche), the Taqman Universal Probe Library, and the Light Cycler Master (Roche) according to the manufacturer’s instructions. Pre-rRNA levels were evaluated by using specific primers for the external transcribed spacer, as described previously [30]. The copy number of mitochondrial DNA was assessed by quantification of a unique mitochondrial DNA fragment relative to a single copy region of the nuclear gene Tfrc (transferrin receptor) using real-time PCR [31].

### Histological Analysis

Liver tissue samples were harvested and fixed in 10% formalin overnight, followed by embedding in paraffin and sectioning. Then the sections were subjected to HE staining or to immunohistochemistry with anti-4-hydroxy-2-nonenal antibody (Abcam).

### Microarray Analysis

The hepatic gene profile of wild-type and *Akt1* ^+/–^ mice was analyzed at 8 weeks and 40 weeks of age by using Agilent Whole Mouse 44K Arrays (n = 3 per group). The raw data were subjected to log2 transformation and normalized by using the GeneSpring GX v7.3.1 (Agilent Technologies). Differentially expressed genes (p<0.01) were determined by two-way ANOVA using two parameters, which were the genetic background (Wild-type or *Akt1* ^+/–^) and the age (8 weeks or 40 weeks). Gene ontology analysis was performed based on each category of two-way ANOVA. Gene expression data were deposited in the Gene Expression Omnibus database (GSE39699).

### Analysis of Mitochondria

Isolation of mitochondria was performed as described previously [32]. In brief, mice were sacrificed by decapitation and their livers were harvested immediately, washed in ice-cold isolation buffer (225 mM mannitol, 75 mM sucrose, 5 mM HEPES, 1 mM EGTA), and minced with a razor blade. Then the tissue was homogenized with a motorized Teflon/glass homogenizer, the homogenate was centrifuged for 5 minutes at 500 × g at 4°C, and the supernatant was collected and re-centrifuged for 5 minutes at 500 × g. The resulting supernatant was then centrifuged for 10 minutes at 8000 × g at 4°C, and the pellet was suspended in isolation buffer. Unless otherwise indicated, all procedures were performed on ice. Protein concentrations were determined by the BCA protein assay (Pierce). Oxygen consumption was measured with an Oxygen Meter (Model 781) and a Mitocell MT200 closed respiration chamber (Strathkelvin Instruments, North Lanarkshire, UK) at 37°C with continuous stirring in respiration buffer (125 mM KCl, 1 mM K_2_HPO_4_, 5 mM MgCl_2_, 25 mM HEPES, 0.2 mM EGTA, and 20 mM mannitol). Mitochondria, pyruvate, and malate (2.5 mM each), 500 nM rotenone, and 5 mM succinate were added sequentially to the buffer. Oxygen consumption by complex I was defined as the rotenone-sensitive component of oxygen consumption in the presence of pyruvate plus malate. Oxygen consumption by complex II was defined as consumption after the addition of succinate minus consumption.

### Isolation of Hepatocytes

Hepatocytes were isolated as described previously [33], [34]. In brief, 40 week-old mice were anesthetized and the abdominal cavity was opened. A 23G needle was introduced into the portal vein, and perfusion was started with Hepatocyte Liver Perfusion Medium (1×) (Gibco) after proximal ligation of the inferior vena cava and transection of it distally. Perfusion was continued until the liver became pale, after which Hepatocyte Liver Perfusion Medium was changed to Hepatocyte Liver Digest Medium (1×) (Gibco). Perfusion with the Digest Medium was continued until the liver became soft. Subsequently, the perfused liver was transferred to a Petri dish containing 10 ml of Hepatocyte Liver Perfusion Medium and hepatocytes were isolated in this medium. For evaluation of the mitochondrial membrane potential, hepatocytes were incubated with William’s medium (1×) (Gibco) containing 100 nM tetramethyl rhodamine methyl ester (Invitrogen) for 40 minutes [35]. For evaluation of the cellular ROS content, hepatocytes were incubated with 5 μM 2,7-dichlorodihydrofluorescein diacetate (Invitrogen) for 60 minutes [36]. Cells were washed 3 times with PBS containing 1% FCS, and then subjected to FACS analysis.

### C. Elegans Culture

*C. elegans* strains were cultured and synchronized as described previously [37]. All strains were maintained at 22°C. The lifespan was investigated as described previously [38], using the L1 period as t = 0 for lifespan analysis. We examined 80–100 nematodes for each condition and performed daily observation. All lifespan analyses were conducted at least twice. RNAi bacterial strains were purchased from the Ahringer library (Source BioScience UK Limited) and the Fire library (Open Biosystems), and were cultured and utilized as described previously [37], [39]. Nematodes at the L4 stage were transferred to RNAi bacterial plates in the presence of 1 mM isopropyl β-D-thiogalactopyranoside (IPTG) and 25 mg/ml carbenicillin, with 5-fluoro-20-deoxyuridine (FUdR, 0.5 mg/ml) being added to prevent the production of progeny. Control nematodes were incubated on plates containing bacteria with the empty RNAi vector. All steps were carried out at 22°C.

## Results

### Haploinsufficiency of *Akt1* Prolongs the Lifespan of Mice

**Figure 1:**
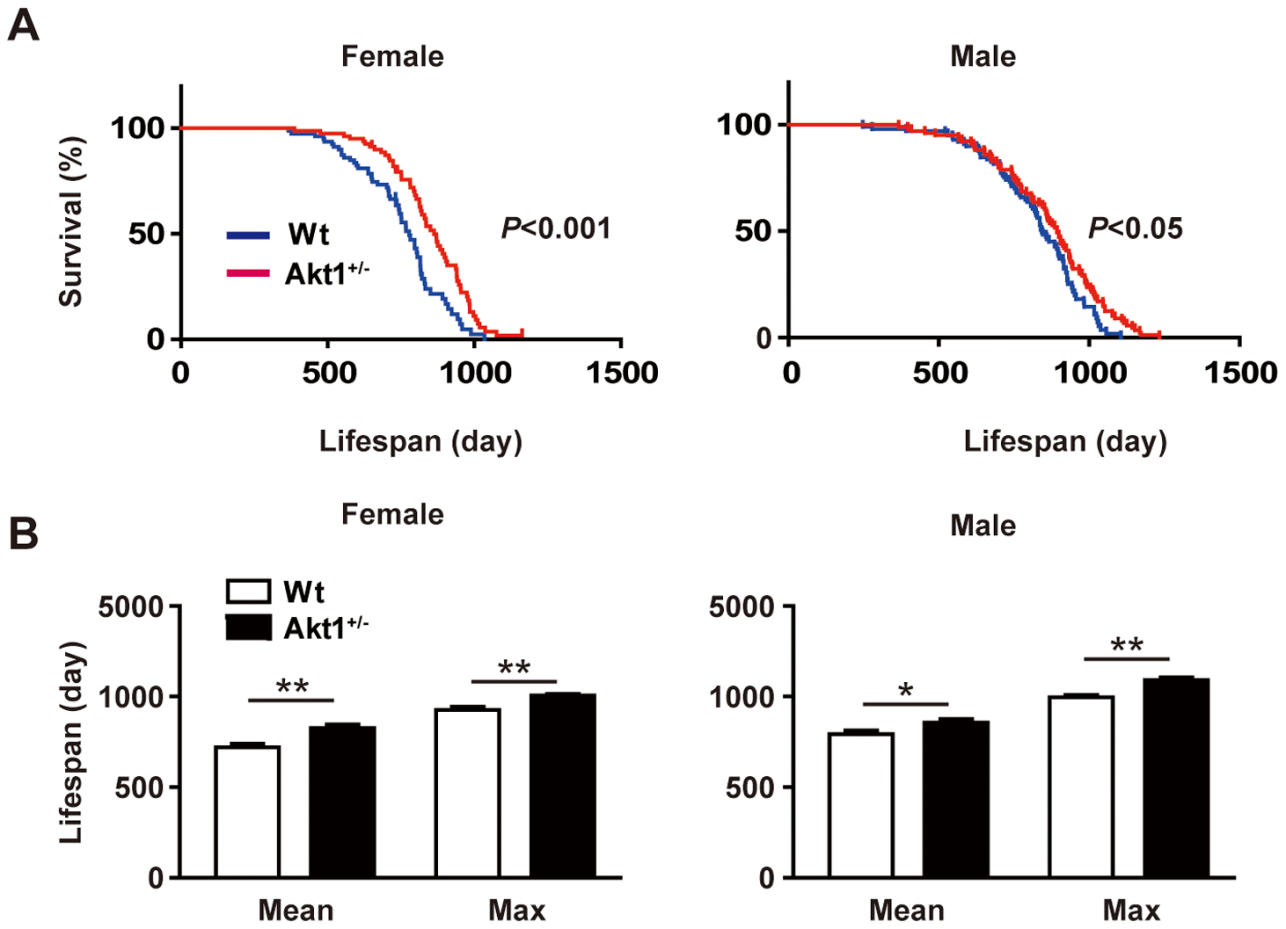
Lifespan of *Akt1* ^+/–^ mice and microarray data. (**A**) Kaplan-Meier survival curves for *Akt1* ^+/–^ mice and wild-type littermates show a significantly longer lifespan of *Akt1* ^+/–^ mice (n = 101 for wild-type male mice, n = 103 for *Akt1* ^+/–^ male mice, n = 79 for wild-type female mice, n = 80 for *Akt1* ^+/–^ female mice). (**B**) The median and maximum lifespan of *Akt1* ^+/–^ mice were significantly increased for both genders. Maximum lifespan was calculated as the average for the oldest 20% of the mice in each group. Mean lifespan (female): 720.7±20.36 vs. 827.2±19.1 days. Maximum lifespan (female): 926.6±17.6 vs. 1005.0±9.1 days. Mean lifespan (male): 793.7±18.8 vs. 857.4±19.1 days. Maximum lifespan (male): 995.7±12.9 vs. 1091.0±14.2 days. Data are shown as the mean ± s.e.m. *P<0.05, **P<0.001. Differences of lifespan between groups were evaluated by the log-rank test.

To investigate the role of the insulin/IGF1 pathway in regulation of the lifespan, we examined the effect of haploinsufficiency of *Akt1*, a gene encoding a key kinase in the insulin/IGF1 signaling pathway, on the lifespan of mice. We utilized *Akt1* ^+/–^ mice because *Akt1* ^–/–^ mice show pathological features such as an increase of apoptosis in various tissues [40], [41]. We found that the level of phospho-Akt1 increased with age in wild-type mice, while this increase was attenuated in *Akt1* ^+/–^ mice (Fig. S1). We compared *Akt1* ^+/–^ mice with their wild-type littermates (on a C57BL/6 background) (n = 363) for 3 years in a blinded study, i.e., the observers were unaware of the genotype of each group of animals. Kaplan-Meier survival analysis of *Akt1* ^+/–^ mice and their wild-type littermates showed that the median lifespan of the former was significantly longer than that of the latter. The difference was larger for female *Akt1* ^+/–^ mice (Fig. 1A, B), but the maximum lifespan of both males and females was significantly longer (Fig. 1A, B).

### Pathophysiological Features of *Akt1* ^+/–^ Mice

**Figure 2:**
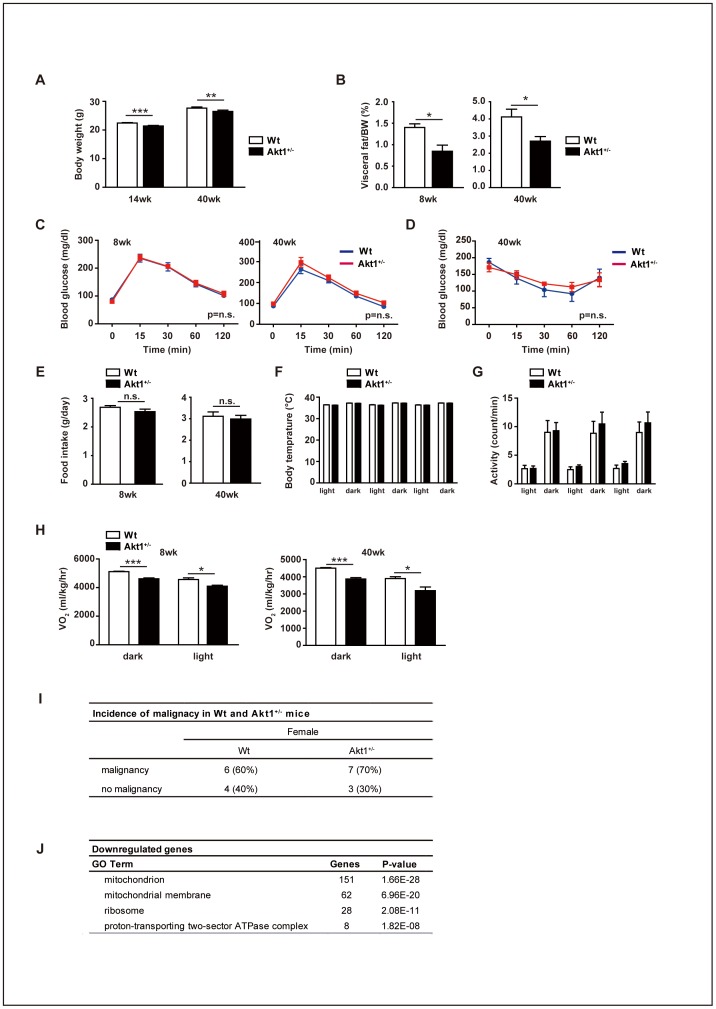
Pathophysiological features of young and middle-aged female *Akt1* ^+/–^ mice. (**A**) Body weight of wild-type and *Akt1* ^+/–^ female mice at 14 weeks and 40 weeks old (n = 58). Body weight was lower in *Akt1* ^+/–^ mice. (**B**) Visceral fat weight per unit body weight of wild-type and *Akt1* ^+/–^ female mice at 8 weeks and 40 weeks old (n = 7). Visceral fat was reduced in *Akt1* ^+/–^ mice. (**C**) Glucose tolerance of wild-type and *Akt1* ^+/–^ female mice at 8 weeks and 40 weeks old (n = 8). Glucose tolerance did not differ between the two strains. (**D**) Insulin tolerance of wild-type and *Akt1* ^+/–^ female mice at 40 weeks old (n = 8). (**E**) Food intake of wild-type and *Akt1* ^+/–^ female mice at 8 weeks and 40 weeks old (n = 8). There were no significant differences between the two strains. (**F**, **G**) Body temperature (**F**) and locomotor activity (G) of wild-type and *Akt1* ^+/–^ female mice at 40 weeks old (n = 8). (H) Oxygen consumption of wild-type and *Akt1* ^+/–^ female mice at 8 weeks and 40 weeks old (n = 5). *Akt1* ^+/–^ mice showed a significant reduction of oxygen consumption. (**I**) Histological examination of wild-type and *Akt1* ^+/–^ female mice at 2 years old (n = 10). The prevalence of malignancy did not differ between the two strains. Data are shown as the mean ± s.e.m. *P<0.05, **P<0.01, and ***P<0.001 by Student's *t*-test for A–H. (**J**) Microarray analysis of liver samples from *Akt1* ^+/–^ female mice and wild-type littermates (n = 3). Gene ontology analysis demonstrated that genes related to the mitochondria and ribosomes were significantly down-regulated in *Akt1* ^+/–^ mice (two-way ANOVA).

We next investigated whether there were any differences of pathophysiological features between *Akt1* ^+/–^ mice and their littermate controls. Consistent with the previous report of *Akt1* ^–/–^ mice [26], *Akt1* ^+/–^ mice had a lower body weight (Fig. 2A). However, the difference of body weight was not significant when corrected by femoral length (Wild-type mice, 1.52±0.04 g/mm; *Akt1* ^+/–^ mice, 1.48±0.04 g/mm; n = 10, p = 0.45). Although CT scanning showed that *Akt1* ^+/–^ mice had less fat mass than their wild-type littermates (Fig. 2B), there were no significant differences of glucose tolerance or insulin tolerance between the two groups (Fig. 2C, D). There were no significant differences of food intake, body temperature, and activity levels between the two groups (Fig. 2E–G). However, the oxygen consumption of *Akt1* ^+/–^ mice was slightly, but significantly, lower than that of their littermates (Fig. 2H). Although *Akt1* ^+/–^mice lived significantly longer than their littermate controls, we did not find any differences of age-associated cardiovascular phenotypes in terms of arterial pressure and cardiac function (Fig. S2A, B). Histological examination of the aorta, bone, and skeletal muscle in aged mice also detected no age-related differences (Fig. S2C).

Since Akt1 signaling has been reported to contribute to tumorigenesis [42], [43], we also investigated the effect of haploinsufficiency of *Akt1* on the development of malignancy. Histological examination of 2-year-old mice demonstrated that the incidence of malignancy was not altered by *Akt1* haploinsufficiency (Fig. 2I). We often observed malignant cells (chiefly lymphomas) infiltrating the tissues (such as liver, skeletal muscle, and visceral fat) of mice over 100 weeks old. Therefore, we used tissue samples from young (8-week-old) and middle-aged mice (40-week-old) mice for further analyses.

### Ribosomal Biogenesis and Mitochondrial Function in *Akt1* ^+/–^ Mice

**Figure 3:**
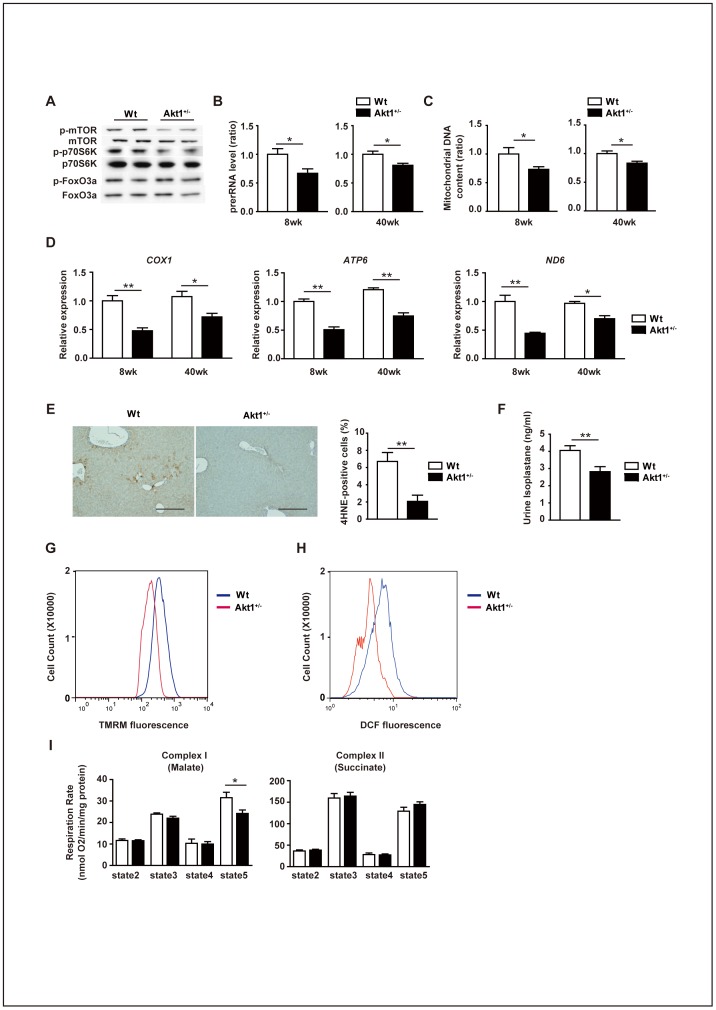
Ribosomal biogenesis and mitochondrial function in young and middle-aged female *Akt1* ^+/–^ mice. (**A**) Western blot analysis of phosphorylated mTOR, phosphorylated p70 S6 kinase, and phosphorylated FoxO3a expression in the livers of wild-type and *Akt1* ^+/–^ mice at 40 weeks old. (**B**) Pre-rRNA level in the livers of wild-type and *Akt1* ^+/–^ mice at 8 weeks and 40 weeks old were examined by real-time PCR (n = 10). (**C**) Mitochondrial DNA content of the livers prepared as Figure 2B. (**D**) Real-time PCR analysis of the expression of *COX1* (encoding cytochrome *c* oxidase subunit I), *ATP6* (encoding ATP synthase Fo subunit 6), and *ND6* (encoding NADH dehydrogenase, subunit 6 (complex I)) in livers prepared as in Fig. 2B (n = 4). (**E**) Immunohistochemistry for 4-hydroxy-2-nonenal (4-HNE) in the livers of wild-type and *Akt1* ^+/–^ mice at 40 weeks old. Scale bar = 100 μm. The right graph displays the quantitative data on 4-HNE-positive cells (n = 6). (**F**) Urinary 8-isoprostane level in wild-type and *Akt1* ^+/–^ mice at 40 weeks old (n = 6). (**G**, **H**) Number of TMRM-positive (G) and DCF-positive (H) hepatocytes isolated from wild-type and *Akt1* ^+/–^ mice at 40 weeks old, as shown by FACS analysis (n = 4). (**I**) Oxygen consumption by complex I (left) and complex II (right) in mitochondria isolated from the livers of wild-type and *Akt1* ^+/–^ mice at 40 weeks old (n = 6). Data are shown as the mean ± s.e.m. *P<0.05, **P<0.01 by Student's *t*-test.

To gain some insight into the potential mechanisms leading to extension of the lifespan in *Akt1* ^+/–^ mice, we performed microarray analysis of liver, skeletal muscle, and visceral fat obtained from these mice and their wild-type littermates. Gene ontology (GO) analysis revealed that mitochondrion and ribosome were among the most significant GO terms (Fig. 2J and Fig. S3). Consistent with these findings, the mTOR pathway, which has a crucial role in regulating ribosomal biogenesis, protein synthesis, and mitochondrial activity [15], [44], was down-regulated in *Akt1* ^+/–^ mice, although phosphorylation of FoxO was unaltered (Fig. 3A and Fig. S4). Indeed, ribosomal biogenesis was markedly reduced in *Akt1* ^+/–^ mice (Fig. 3B), along with a decrease of the mitochondrial DNA content and reduced expression of genes for mitochondrial components and transcription factors involved in mitochondrial biogenesis, when compared with their wild-type littermates (Fig. 3C, D and Fig. S5). These changes were associated with reduced oxidative stress, as demonstrated by a decrease of 4-hydroxynonenal immunoreactivity in the liver and a decline of 8-isoprostane excretion in the urine (Fig. 3E, F), suggesting that partial inhibition of Akt1 activity ameliorated the age-associated increase of oxidative stress.

To further investigate the effects of *Akt1* haploinsufficiency on mitochondrial function and oxidative stress, we isolated hepatocytes from *Akt1* ^+/–^ mice and their littermate controls. Then these cells were subjected to FACS analysis to detect tetramethylrhodamine, methyl ester (TMRM), and dichlorodihydrofluorescein (DCF) fluorescence. This revealed that hepatocytes from *Akt1* ^+/–^ mice showed a significant decrease of the mitochondrial membrane potential and levels of reactive oxygen species (ROS) compared with hepatocytes from their wild-type littermates (Fig. 3G, H). We next examined the activity of mitochondria isolated from the hepatocytes of *Akt1* ^+/–^ mice and their littermate controls, and found that the maximum respiration rate of isolated mitochondria did not differ between the two groups (Fig. 3I). These results indicated that the amounts of mitochondria were decreased in *Akt1* ^+/–^ mice compared with their littermate controls, but the activity of per mg mitochondrion was not impaired by haploinsufficiency of *Akt1*. We noted that the expression of FoxO-regulated antioxidant genes, such as catalase and superoxide dismutase, did not differ between *Akt1* ^+/–^ mice and their littermate controls (Fig. S6). Since ROS are a byproduct of normal mitochondrial respiration, a decrease in the number of mitochondria could account for a decrease of oxidative stress in *Akt1* ^+/–^ mice.

### Ribosomal Biogenesis and Mitochondrial Function in Human Cells and *C. elegans*

**Figure 4:**
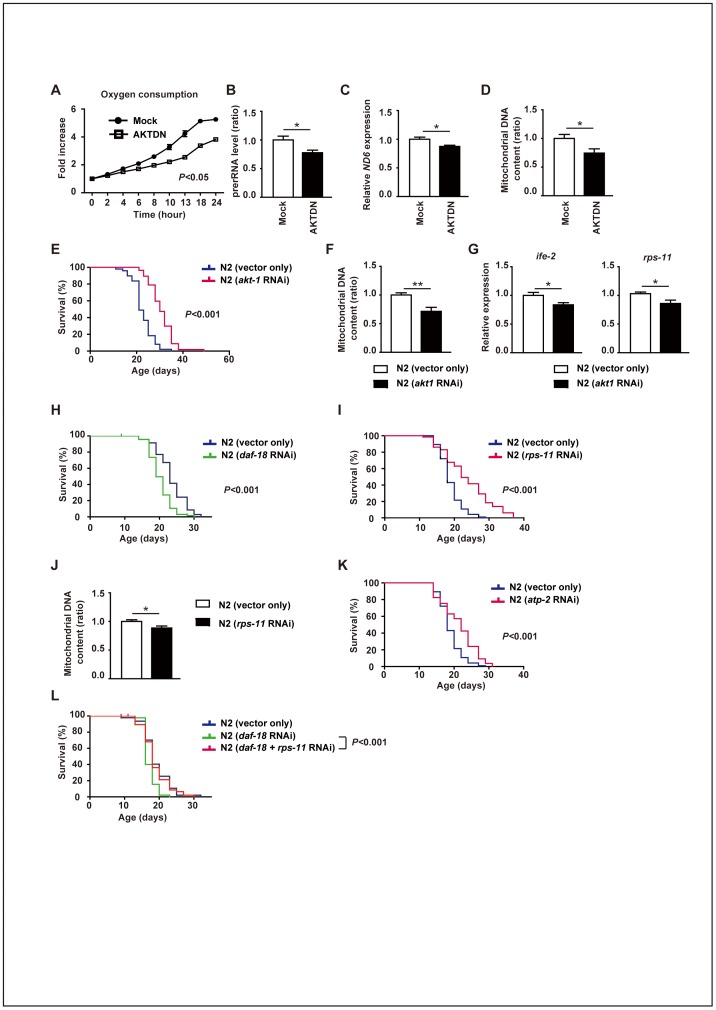
Ribosomal biogenesis and mitochondrial function in human cells and *C. elegans*. (**A**) Oxygen consumption in human endothelial cells infected with a retroviral vector encoding a dominant-negative form of AKT1 (AKTDN) or an empty vector (Mock). Data represent the fold increase relative to the initial value (n = 3). P<0.05 by two-way ANOVA. (**B**) Pre-rRNA level in human endothelial cells infected with AKTDN or Mock (n = 6). (**C**) Real-time PCR analysis of the expression of ND6 (encoding NADH dehydrogenase, subunit 6 (complex I)) in human endothelial cells infected with AKTDN or Mock (n = 6). (**D**) Mitochondrial DNA content of human endothelial cells infected with AKTDN or Mock assessed by real-time PCR (n = 3). Data are shown as the mean ± s.e.m. *P<0.05 by Student's *t*-test. (**E**) Survival of *C. elegans* fed with bacteria containing a control vector or the dsRNA construct targeting *akt-1*. Knockdown of *akt-1* significantly prolonged the lifespan. P<0.001 by the log-rank test. (F) Mitochondrial DNA content in *C. elegans* prepared as in Fig. 4E (n = 8). (**G**) Expression of *ife-2* (encoding translation initiation factor 4F, cap-binding subunit (eIF4E)) and *rps-11* (encoding a small ribosomal subunit S11 protein) in *C. elegans* prepared as in Fig. 3e (n = 8). Data are shown as the mean ± s.e.m. *P<0.05, **P<0.01 by Student's *t*-test. (**H**) Activation of AKT-1 by knockdown of *daf-18* (a homologue of PTEN) led to significant shortening of the lifespan of wild-type worms. P<0.001 by the log-rank test. (**I**) Knockdown of *rps-11* by RNAi led to significant prolongation of the lifespan of wild-type worms. P<0.001 by the log-rank test. (**J**) Mitochondrial DNA content in *C. elegans* prepared as in Fig. 4I (n = 5). Data are shown as the mean ± s.e.m. *P<0.05 by Student's *t*-test. (**K**) Knockdown of *atp-2* (encoding a subunit of ATP synthase, mitochondrial complex V) by RNAi significantly prolonged the lifespan of wild-type worms. P<0.001 by the log-rank test. (**L**) Knockdown of *rps-11* by RNAi improved shortening of the lifespan of worms with *daf-18* RNAi treatment.

To investigate the role of AKT1 in ribosomal biogenesis and in total mitochondrial activity, we infected human endothelial cells with a retroviral vector encoding a dominant-negative form of AKT1. Consistent with the results obtained in *Akt1* ^+/–^ mice, inhibition of AKT1 activity led to a decrease of oxygen consumption and ribosomal biogenesis compared with mock-infected cells (Fig. 4A, B). The mitochondrial DNA content and the expression of genes for mitochondrial components were also reduced by inhibition of AKT1 (Fig. 4C, D).

To gain further insights into the influence of Akt1 on longevity, we examined the influence of inhibiting AKT-1 on ribosomal biogenesis, the mitochondrial DNA content, and the lifespan of *C. elegans*. In agreement with the results obtained in *Akt1* ^+/–^ mice, inactivation of AKT-1 by RNAi resulted in a longer lifespan compared with that of wild-type (N2) *C. elegans* (Fig. 4E), and this change was associated with a decrease of ribosomal gene expression and reduction of the mitochondrial DNA content (Fig. 4F, G). Conversely, activation of AKT-1 by RNAi targeting *daf-18* led to a shorter lifespan (Fig. 4H). Inhibition of ribosomal biogenesis by RNAi decreased the mitochondrial DNA content and extended the lifespan of wild-type animals (Fig. 4I, J), while inhibition of mitochondrial function by RNAi also increased the lifespan of wild-type animals (Fig. 4K). Moreover, inhibition of ribosomal biogenesis normalized the shortened lifespan of nematodes treated with *daf-18* RNAi (Fig. 4L), suggesting that the decrease of ribosomal biogenesis and mitochondrial function were critical for improving the longevity of *Akt1* ^+/–^ mice.

## Discussion

The present study demonstrated that haploinsufficiency of *Akt1* significantly prolongs the lifespan of mice. *Akt1* ^+/–^ mice showed a decrease of ribosomal biogenesis and the amounts of mitochondria, which was associated with a reduction of oxidative stress. It has been reported that the insulin/IGF1 signaling pathway and the TOR pathway both promote ribosomal biogenesis and protein synthesis, as well as regulating mitochondrial function [15], [44]. We found that the phospho-Akt1 level increased with age in wild-type mice, presumably due to constitutive activation of growth signals by various stresses. Given the large amount of energy consumed by ribosomal biogenesis and protein synthesis, *Akt1* ^+/–^ mice may utilize fewer mitochondria by reducing ribosomal biogenesis and thus minimize the enhancement of oxidative stress with aging, which could account for their longer lifespan. Decreased protein synthesis could improve the fidelity of biogenesis and prevent the accumulation of mis-folded proteins, which might also contribute to longevity associated with *Akt1* deficiency. Since oxidative stress and oxygen consumption were both lower in *Akt1* ^+/–^ mice than in their littermates, an increase of mitochondria and energy expenditure associated with enhanced protein synthesis may accelerate aging and promote the onset of age-associated diseases.

An intriguing aspect of longevity pathways is that they function independently of the cells affected, since mutations of one cell type can affect the phenotype of whole organism. Neuronal cells and adipose tissue have been suggested to have a critical role in regulation of the lifespan by the insulin/IGF1 pathway in nematodes and flies [10], [45]. For example, increasing the level of DAF-16 expression in one tissue can increase DAF-16 activity elsewhere, through feedback regulation of insulin gene expression [46]. In addition, in *daf-16* (–); *daf-2* (–) double mutant nematodes (short-lived animals), overexpression of DAF-16 in one tissue can increase the lifespan [47]. Inhibition of the insulin/IGF1 pathway in the brain or adipose tissue has been shown to induce longevity in mice [14], [48], suggesting that lifespan can be regulated cell non-autonomously in mammals. In the present study, we did not determine the tissue(s) responsible for longevity in *Akt1* ^+/–^ mice. Thus, it would be interesting to test the effects of tissue-specific deletion of *Akt1* on the lifespan in the future.

Consistent with our findings, modest inhibition of respiration has been reported to prolong the lifespan of a variety of species, such as yeast, nematodes, flies, and mice [49]–[52]. This increase of longevity could be partly attributable to reduction of the metabolic rate in these animals. In contrast, increasing respiration was reported to promote longevity in animals with caloric restriction [53], [54], so it is possible that increasing or reducing respiration can influence the lifespan in various ways.

Genetic inhibition of autophagy induces degenerative changes in mammalian tissues that resemble those associated with aging, while normal and pathological aging are often associated with a reduced autophagic potential [15], [55]. Genetic manipulations that prolong the lifespan in various models often stimulate autophagy, and inhibition of autophagy compromises the longevity-promoting effect of calorie restriction or suppression of insulin/insulin growth factor signaling [15], [55]. Since mTOR is a primordial negative regulator of autophagy, an increase of autophagic activity may also contribute to extending the lifespan of *Akt* ^+/–^ mice. In this context, it would be interesting to examine the effect of inhibiting the TOR/autophagy pathway on the lifespan of *C. elegans* with *akt-1* or *daf-18* knockdown.

Telomeres are specialized DNA-protein structures found at the ends of eukaryotic chromosomes that serve as markers of biological aging [56]. Telomeres also play a critical role in maintaining genomic integrity and are involved in age-related diseases [28], [57]. Shortening of telomeres is hazardous to healthy cells, as it is a known mechanism of premature cellular senescence and reduction of longevity. Telomerase is an enzyme that adds telomeres to the ends of chromosomes. Although the insulin/Akt pathway has been reported to positively regulate telomerase activity [58], mice have high telomerase activity and long telomeres [59], [60]. Therefore, it is unlikely that Akt1 signaling regulates longevity by modulating telomerase activity in mice.

In conclusion, our results suggest that haploinsufficiency of *Akt1* significantly promotes longevity in mice by mechanisms that involve reduction of both energy expenditure and oxidative stress. Further studies on improvement of longevity related to inhibition of the insulin/IGF-1 pathway should provide useful insights into the treatment of diseases associated with aging.

## Supporting Information

Figure S1 **Age-associated increase of phospho-Akt1 expression.** Western blot analysis of phosphorylated Akt1 expression in the livers of wild-type (Wt) and *Akt1* ^+/–^ female mice at 8 and 40 weeks old.
(DOCX)

Figure S2 **Examination of age-related phenotypes.** (A) Arterial pressure of wild-type (Wt) and *Akt1* ^+/–^ female mice at 100 weeks old. Data are shown as the means ± s.e.m. (B) Echocardiographic analysis of wild-type (Wt) and *Akt1* ^+/–^ female mice at 100 weeks old. FS, fractional shortening; LVDs, left ventricular diastolic dimension. Data are shown as the means ± s.e.m. (C) Hematoxylin-eosin staining of the aorta, bone, and skeletal muscle of wild-type (Wt) and *Akt1^+/–^* female mice at 100 weeks old. Scale bar: 20 μm.
(DOCX)

Figure S3 **Microarray analysis.**
Microarray analysis of fat and skeletal muscle samples from *Akt1* ^+/–^ female mice and wild-type littermates (n = 3).
(DOCX)

Figure S4 **Expression of phopho-FoxO.**
Western blot analysis of phosphorylated FoxO3a expression in various tissues of wild-type (Wt) and *Akt1* ^+/–^ female mice at 100 weeks old.
(DOCX)

Figure S5 **Expression of transcription factors involved in mitochondrial biogenesis.**
The expression of Pgc-1α (also known as *Ppargac1a*) and its regulating molecules related to mitochondrial biogenesis, such as *nuclear respiratory factor* (*Nrf*)*-1* and *mitochondrial transcription factor A* (*Tfam*) was examined by real-time PCR in livers of wild-type (Wt) and *Akt1* ^+/–^ female mice at 40 weeks old. Data are shown as the mean ± s.e.m (n = 5–8). *P<0.05.
(DOCX)

Figure S6 **Expression of antioxidant genes.**
The expression of catalase (*Cat*) and superoxide dismutase 2 (*Sod2*) was examined by real-time PCR in livers of wild-type (Wt) and *Akt1* ^+/–^ female mice at 100 weeks old. Data are shown as the mean ± s.e.m (n = 4). *P<0.05.
(DOCX)
