# Supplementary material for: Haploinsufficiency of Akt1 Prolongs the Lifespan of Mice
Source: PLoS One. 2013 Jul 30;8(7):e69178. doi: 10.1371/journal.pone.0069178 (PMC3728301; doi:10.1371/journal.pone.0069178)
Supplement: Figure S2 — Examination of age-related phenotypes. (A) Arterial pressure of wild-type (Wt) and Akt1 +/– female mice at 100 weeks old. Data are shown as the means ± s.e.m. (B) Echocardiographic analysis of wild-type (Wt) and Akt1 +/– female mice at 100 weeks old. FS, fractional shortening; LVDs, left ventricular diastolic dimension. Data are shown as the means ± s.e.m. (C) Hematoxylin-eosin staining of the aorta, bone, and skeletal muscle of wild-type (Wt) and Akt1+/– female mice at 100 weeks old. Scale bar: 20 μm. (DOCX) [file pone.0069178.s002.docx]

**Supplementary Figure 2**

**
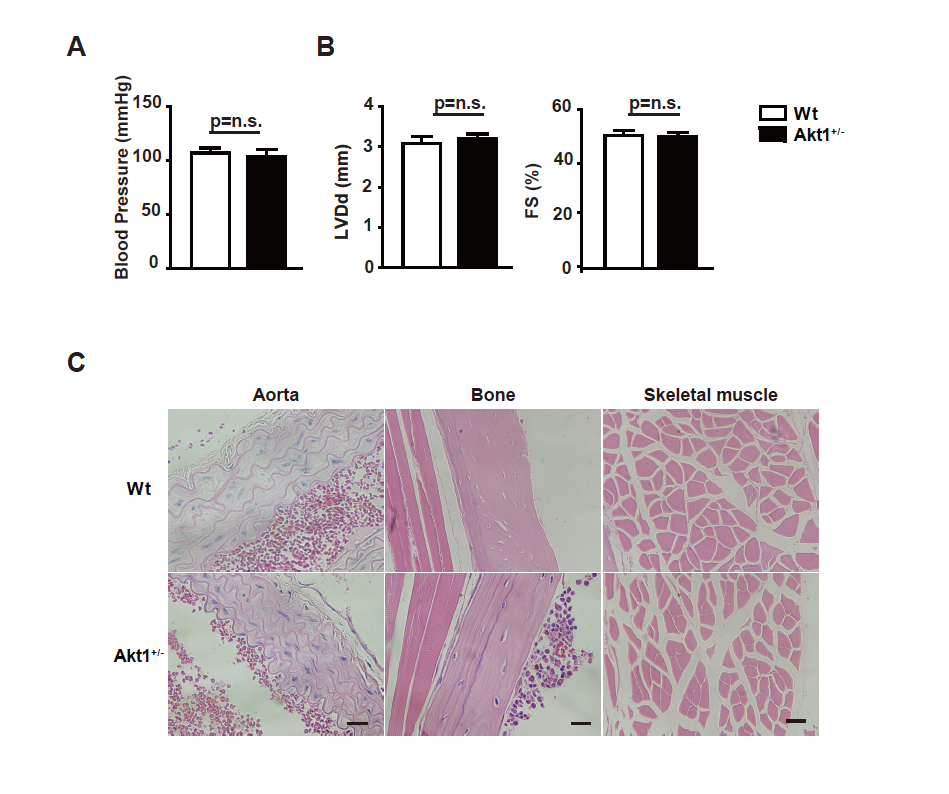
**

**Examination of age-related phenotypes**

(A) Arterial pressure of wild-type (Wt) and *Akt1*^+/–^ female mice at 100 weeks old. Data are shown as the means ± s.e.m.

(B) Echocardiographic analysis of wild-type (Wt) and *Akt1*^+/–^ female mice at 100 weeks old. FS, fractional shortening; LVDs, left ventricular diastolic dimension. Data are shown as the means ± s.e.m.

(C) Hematoxylin-eosin staining of the aorta, bone, and skeletal muscle of wild-type (Wt) and *Akt1^+/–^* female mice at 100 weeks old. Scale bar: 20 μm.
