# Supplementary material for: Haploinsufficiency of Akt1 Prolongs the Lifespan of Mice
Source: PLoS One. 2013 Jul 30;8(7):e69178. doi: 10.1371/journal.pone.0069178 (PMC3728301; doi:10.1371/journal.pone.0069178)
Supplement: Figure S3 — Microarray analysis. Microarray analysis of fat and skeletal muscle samples from Akt1 +/– female mice and wild-type littermates (n = 3). (DOCX) [file pone.0069178.s003.docx]

**Supplementary Figure 3**

**
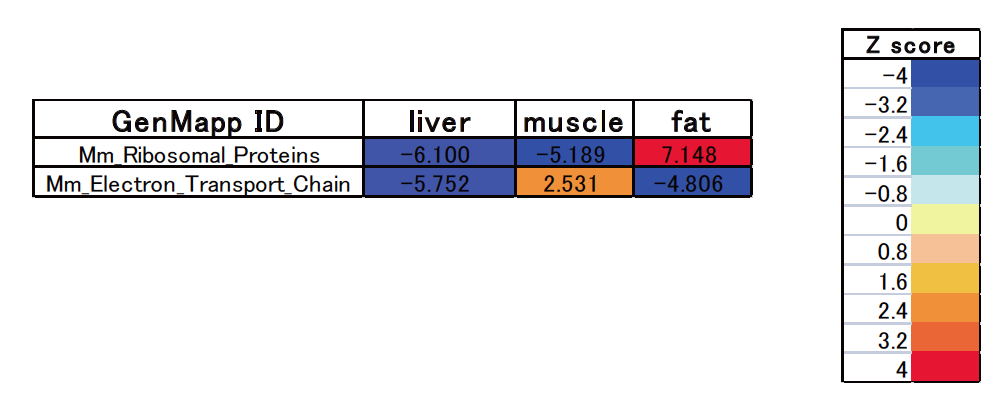
**

**Microarray analysis**

The parametric analysis of gene set enrichment (PAGE) of liver, muscle and fat tissues from wild-type and *Akt1*^+/–^ female mice at 40 weeks old (n=3) detected Ribosomal_Proteins and Electron_Transport_Chain as the significantly changed gene sets.
