# Supplementary material for: Haploinsufficiency of Akt1 Prolongs the Lifespan of Mice
Source: PLoS One. 2013 Jul 30;8(7):e69178. doi: 10.1371/journal.pone.0069178 (PMC3728301; doi:10.1371/journal.pone.0069178)
Supplement: Figure S4 — Expression of phopho-FoxO. Western blot analysis of phosphorylated FoxO3a expression in various tissues of wild-type (Wt) and Akt1 +/– female mice at 100 weeks old. (DOCX) [file pone.0069178.s004.docx]

**Supplementary Figure 4**

**
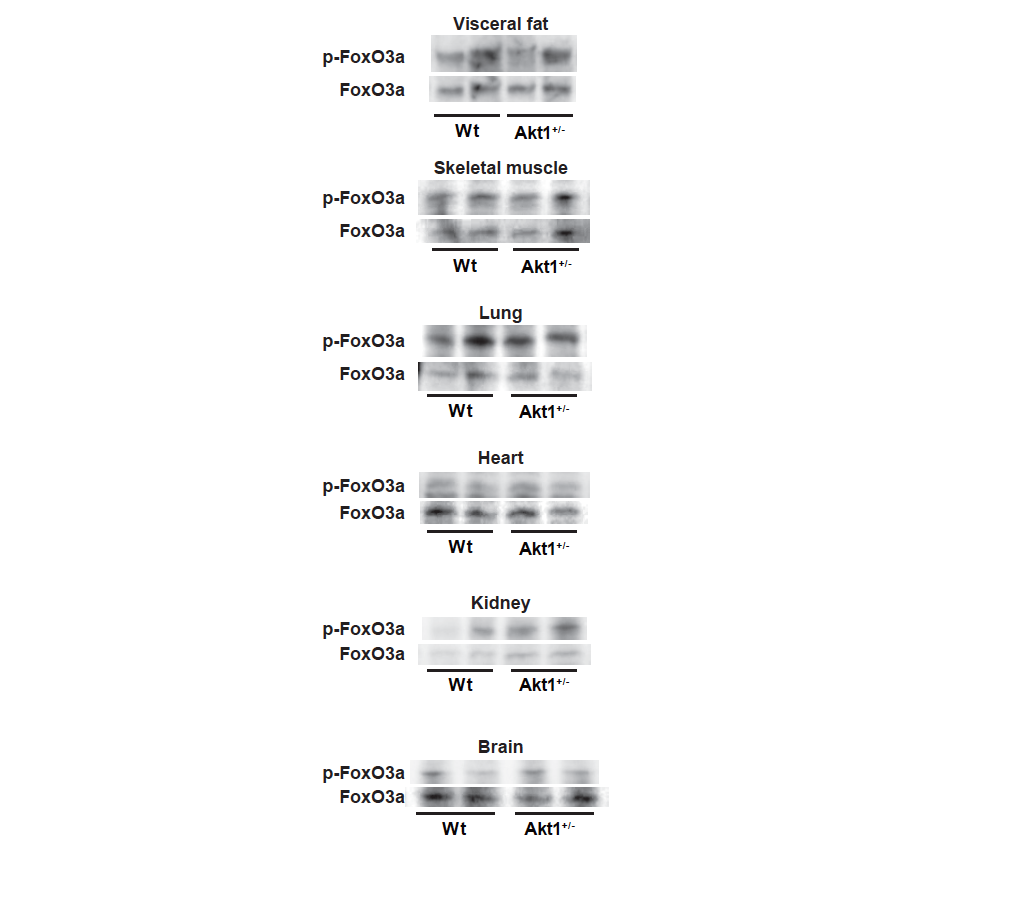
**

**Expression of phopho-FoxO**

Western blot analysis of phosphorylated FoxO3a expression in various tissues of wild-type (Wt) and *Akt1*^+/–^ female mice at 100 weeks old.
