# Supplementary material for: Haploinsufficiency of Akt1 Prolongs the Lifespan of Mice
Source: PLoS One. 2013 Jul 30;8(7):e69178. doi: 10.1371/journal.pone.0069178 (PMC3728301; doi:10.1371/journal.pone.0069178)
Supplement: Figure S5 — Expression of transcription factors involved in mitochondrial biogenesis. The expression of Pgc-1α (also known as Ppargac1a) and its regulating molecules related to mitochondrial biogenesis, such as nuclear respiratory factor (Nrf)-1 and mitochondrial transcription factor A (Tfam) was examined by real-time PCR in livers of wild-type (Wt) and Akt1 +/– female mice at 40 weeks old. Data are shown as the mean ± s.e.m (n = 5–8). *P<0.05. (DOCX) [file pone.0069178.s005.docx]

**Supplementary Figure 5**

**
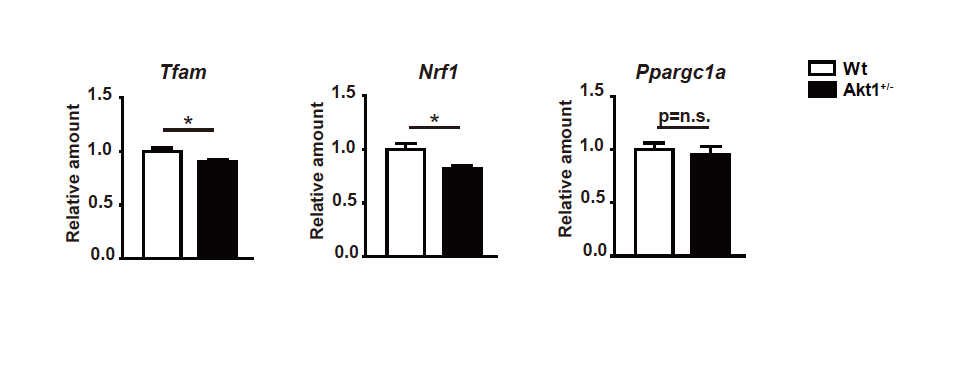
**

**Expression of transcription factors involved in mitochondrial biogenesis**

The expression of Pgc-1α (also known as *Ppargac1a*) and its regulating molecules related to mitochondrial biogenesis, such as *nuclear respiratory factor* (*Nrf*)*-1* and *mitochondrial transcription factor A* (*Tfam*) was examined by real-time PCR in livers of wild-type (Wt) and *Akt1*^+/–^ female mice at 40 weeks old. Data are shown as the mean ± s.e.m (n=5–8). *P<0.05.
