# Supplementary material for: Haploinsufficiency of Akt1 Prolongs the Lifespan of Mice
Source: PLoS One. 2013 Jul 30;8(7):e69178. doi: 10.1371/journal.pone.0069178 (PMC3728301; doi:10.1371/journal.pone.0069178)
Supplement: Figure S6 — Expression of antioxidant genes. The expression of catalase (Cat) and superoxide dismutase 2 (Sod2) was examined by real-time PCR in livers of wild-type (Wt) and Akt1 +/– female mice at 100 weeks old. Data are shown as the mean ± s.e.m (n = 4). *P<0.05. (DOCX) [file pone.0069178.s006.docx]

**Supplementary Figure 6**

**
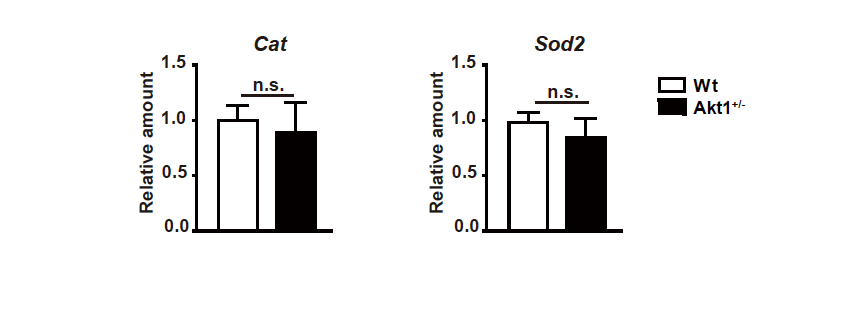
**

**Expression of antioxidant genes**

The expression of catalase (*Cat*) and superoxide dismutase 2 (*Sod2*) was examined by real-time PCR in livers of wild-type (Wt) and *Akt1*^+/–^ female mice at 40 weeks old. Data are shown as the mean ± s.e.m (n=4). *P<0.05.
